# Abdominal Ectopic Pregnancy: A Case Report of an Uncommon Complication of Pregnancy Without Prenatal Care

**DOI:** 10.7759/cureus.68478

**Published:** 2024-09-02

**Authors:** James A Durden, Samuel L Burleson, David C Pigott, John P Gullett, Maxwell Thompson

**Affiliations:** 1 Emergency Medicine, University of Alabama at Birmingham School of Medicine, Birmingham, USA

**Keywords:** ct (computed tomography) imaging, emergency medicine imaging, abdominal ectopic pregnancy, sonography in the diagnosis of ectopic pregnancy, point-of-care-ultrasound

## Abstract

Ectopic pregnancy (EP) is a life-threatening condition requiring a high clinical suspicion. This diagnosis must be considered in all female patients of reproductive age presenting with abdominal pain or discomfort who may possibly be pregnant. Ectopic pregnancies occur in a small percentage of all pregnancies and are a significant cause of maternal morbidity and mortality. Abdominal ectopic pregnancy (AEP) is a rare and potentially fatal form of ectopic pregnancy where the implantation occurs in the abdominal cavity. We present the following case of a 23-year-old female who was transferred following an initial workup for abdominal pain and subsequently found to have an abdominal ectopic pregnancy at 37 weeks gestation. After transferring to our emergency department, the patient continued to have abdominal pain and her presenting FAST exam was positive for free fluid concerning for active hemorrhage and hematoma. Her clinical presentation was consistent with ruptured abdominal ectopic pregnancy, and she was taken to the operating room for emergent exploratory laparotomy and delivery. Her clinical course was complicated by adherent placenta and re-bleeding with significant hemoperitoneum requiring re-entry laparotomy and transfusion. We present the details of this case along with the diagnostic imaging and management of the rarely seen and life-threatening condition of secondary abdominal ectopic pregnancy (AEP).

## Introduction

Abdominal ectopic pregnancy (AEP) is a rare, potentially fatal form of ectopic pregnancy (EP) that can arise either primarily or secondarily [1,2]. The most common sites of ectopic implantation are ampullary (70%), isthmic (12%), fimbrial (11.1%), and ovarian (3.2%) [3]. Primary AEP occurs when the ovum is initially fertilized in the abdominal cavity while the more common secondary AEP arises when the abdomen is the secondary site of implantation [2]. This can occur after a missed, aborted, or ruptured tubal ectopic pregnancy or a change in implantation site due to a uteroplacental fistula or rupture [2]. In AEP, the placenta attaches to the mesentery, uterine wall, or bowel, and has sufficient blood supply to progress to a full-term and possibly viable pregnancy. The maternal and fetal risk, however, is high due to inevitable abruption and hemorrhage [4]. The blood loss can be severe, as the placenta in AEP attaches to noncontractile tissue, unlike the uterus, which can contract following placental separation, reducing postpartum bleeding [5]. Ultrasound is the imaging modality of choice when evaluating for EP [5,6] and with appropriate prenatal care, many ectopic pregnancies are identified in early pregnancy. Once a diagnosis of AEP is made, magnetic resonance imaging may also be used for surgical planning to identify the placental insertion site and to evaluate for solid organ, mesenteric, and uterine involvement [6].

## Case presentation

**Figure 1:**
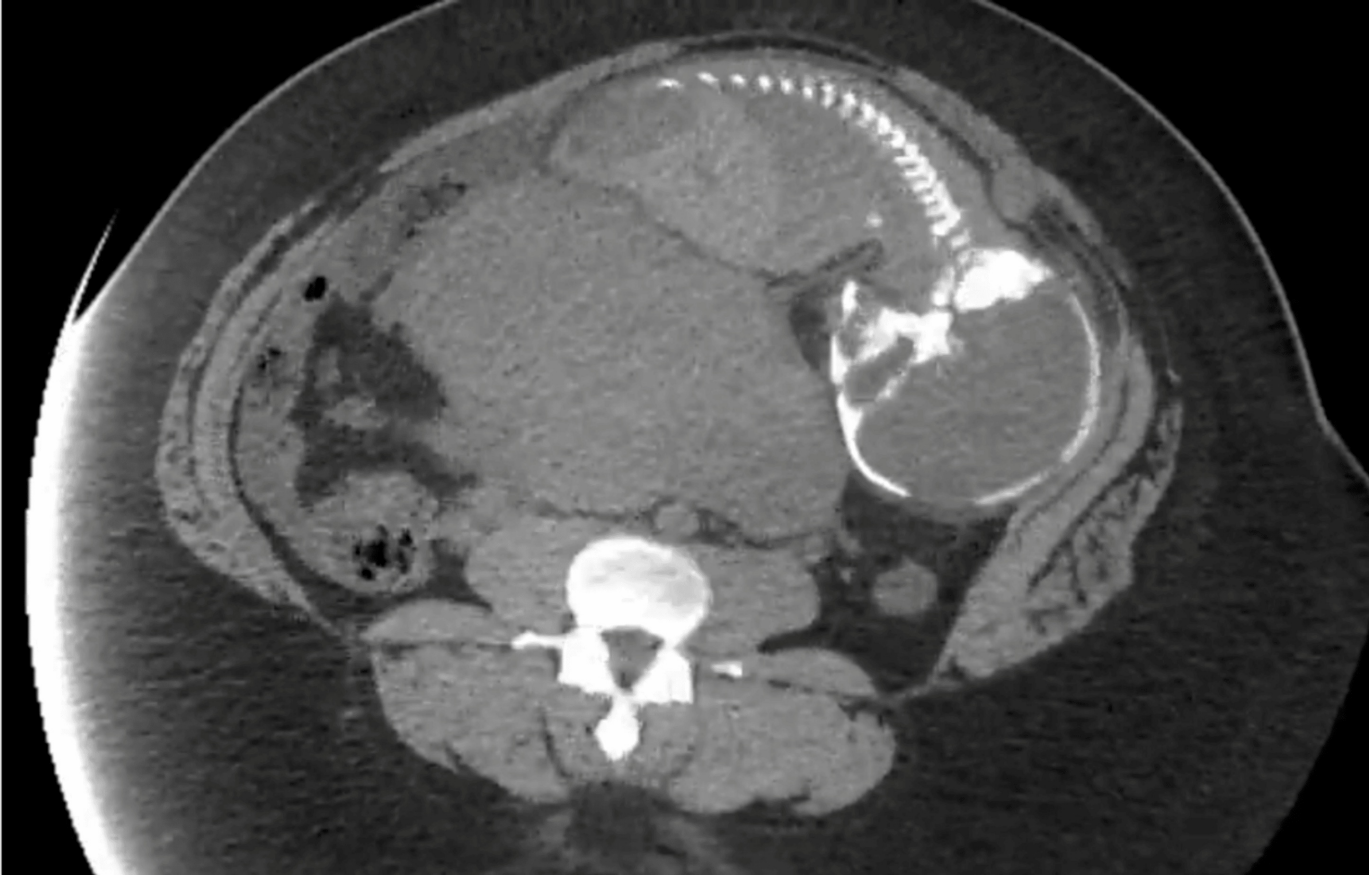
Noncontrasted CT scan showing an intraperitoneal fetus

A 23-year-old female with a past medical history of iron deficiency anemia presented to an outside emergency department with 24 hours of abdominal pain. She was transferred to our facility for obstetric specialty services due to concern for ectopic pregnancy. The patient presented to the outside facility with severe, significant epigastric abdominal pain and peritonitis. She had no other associated symptoms such as nausea, vomiting, or vaginal bleeding. Due to her presentation and peritonitis, an emergent noncontrasted CT of the abdomen and pelvis was performed prior to routine screening for pregnancy as the differential for peritonitis is broad and includes many surgical emergencies such as perforation, obstruction, or infection. Initial imaging at the outside facility consisted of an ultrasound and a non-contrasted computed tomography (CT) scan of the abdomen and pelvis which was concerning for AEP (Figure 1). The patient was G3, P2002 with no prior obstetric complications and was estimated to be at 37 weeks gestation by the last menstrual period, but had not received prenatal care or previous imaging. 

**Figure 2:**
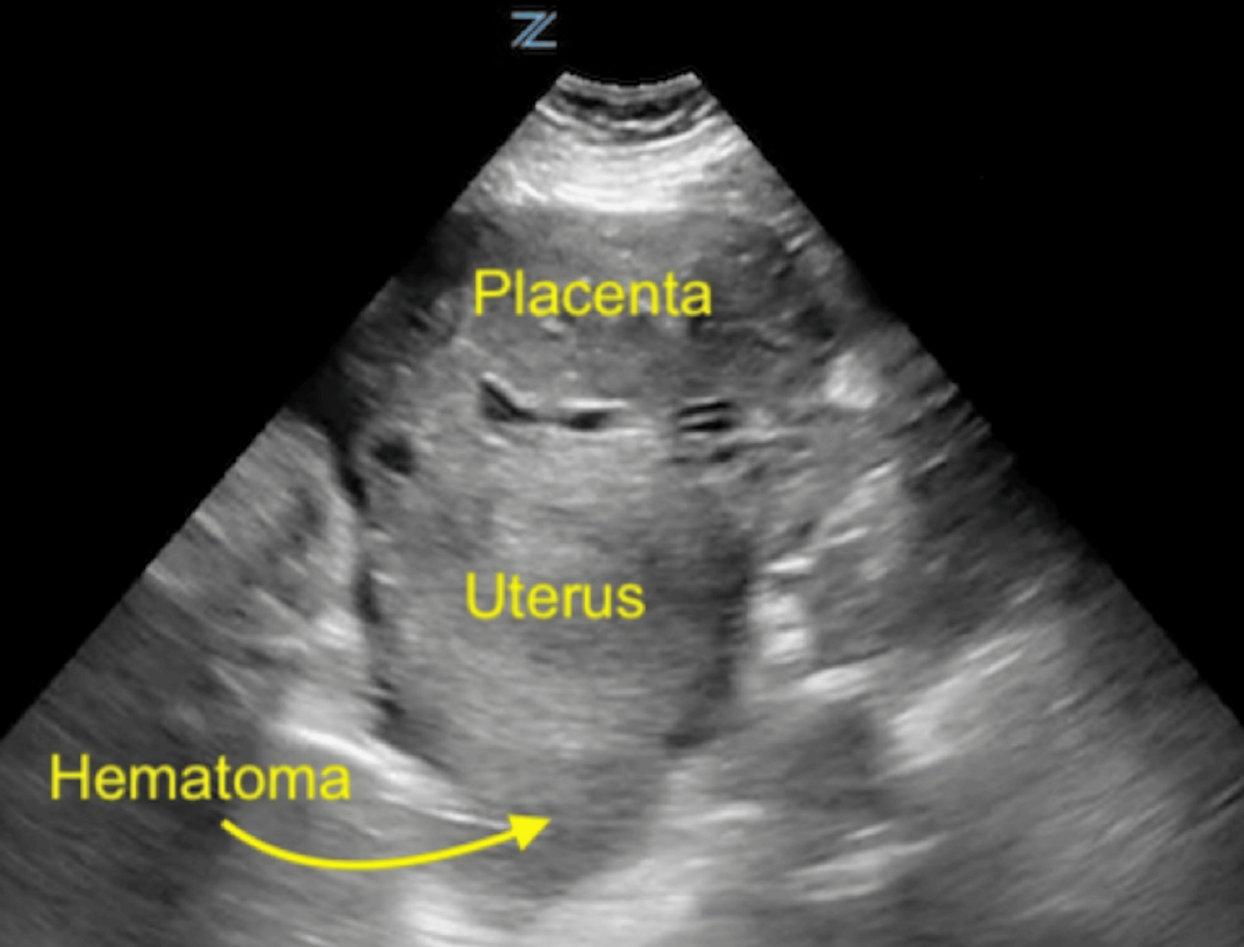
Point-of-care ultrasound in the emergency department showing the uterus and placenta in the axial plane with associated hematoma posterior to the uterus

**Figure 3:**
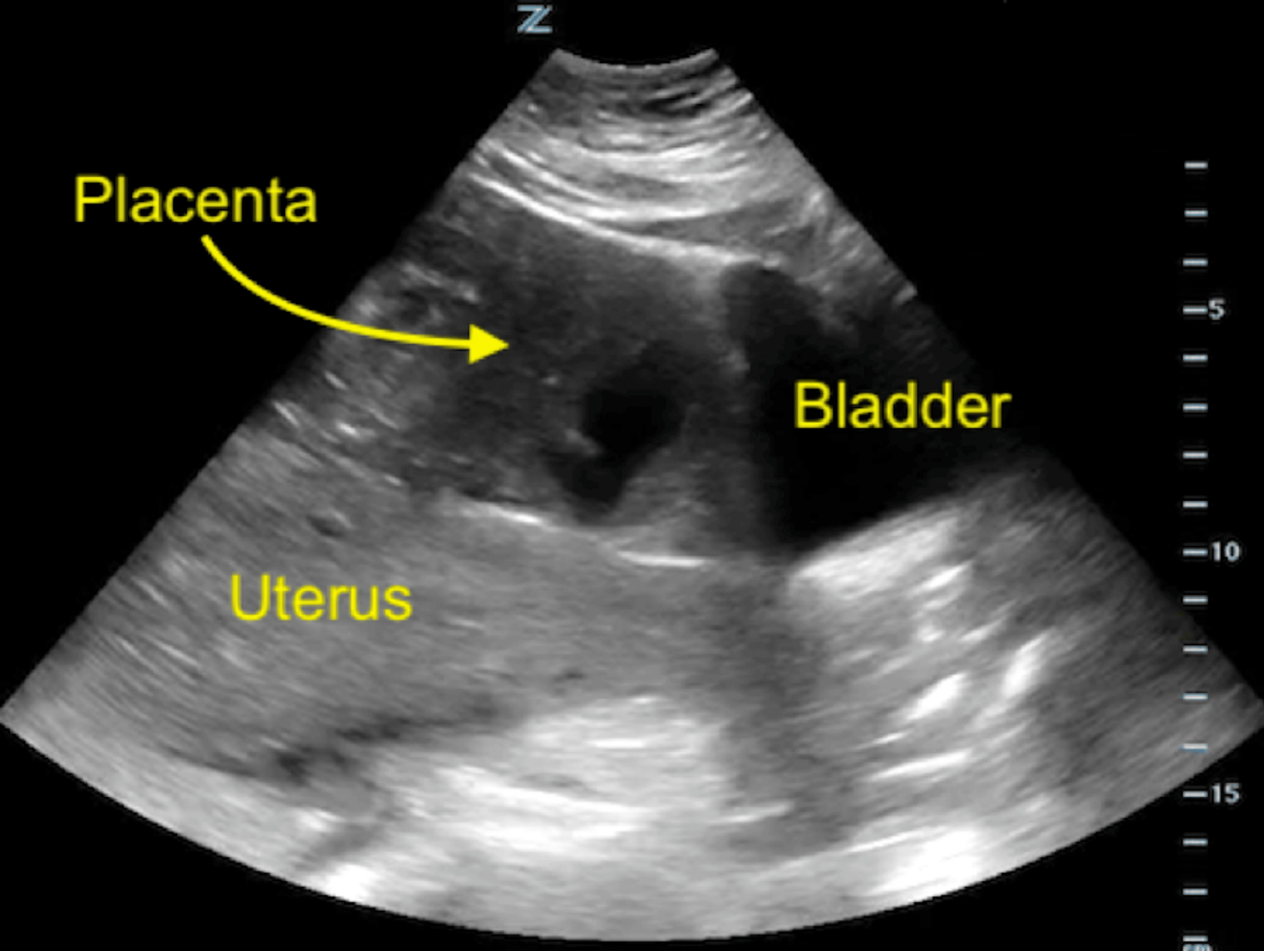
Point-of-care ultrasound in the emergency department showing the uterus and placenta in the sagittal plane

These imaging findings prompted a transfer to our hospital for obstetric specialty care. On arrival to our emergency department, the patient had a blood pressure of 120 mmHg/80 mmHg, a heart rate of 112 bpm, respirations of 18, and a room air oxygen saturation of 99%. She continued to complain of severe abdominal pain and she was given intravenous fentanyl to address this. The patient's initial physical exam was significant for a distended gravid abdomen with peritoneal signs, including voluntary guarding and rebound tenderness. A bedside focused assessment with sonography in trauma (FAST) exam was performed and was positive for free intraperitoneal fluid in the right upper quadrant (RUQ) and a single extrauterine pregnancy with a fetal heart rate of 153. The placenta was visualized external to and overlying the uterus (Figures 2, 3). Her presentation was highly concerning for active hemorrhage. After an emergency obstetric consult, the patient was taken emergently to the operating room (OR) for exploratory laparotomy and delivery. 

**Figure 4:**
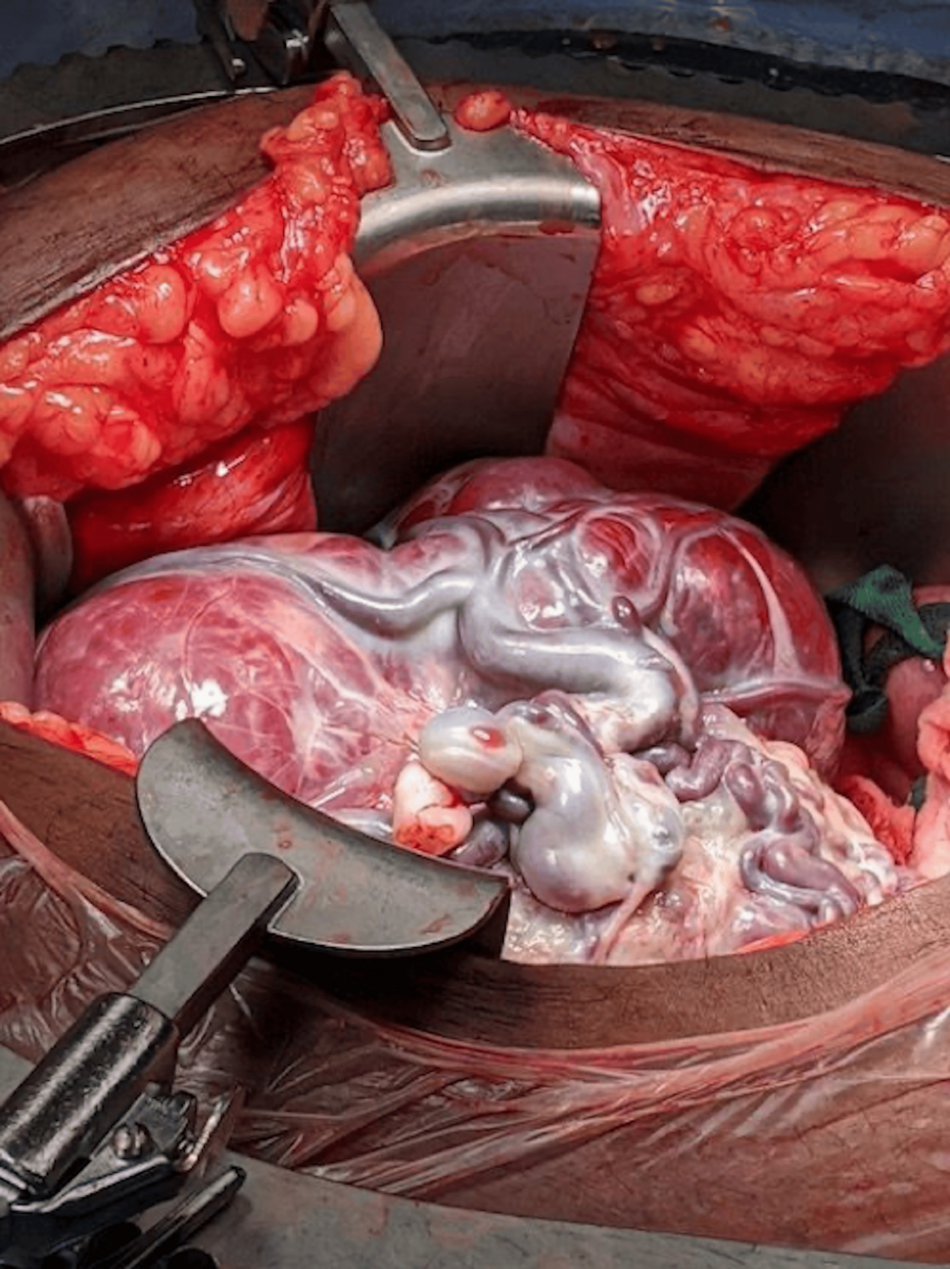
Intrabdominal findings of placenta in the peritoneal space

Intraoperatively, she was found to have the placenta densely adhered to the uterine fundus with a ruptured uterine horn but normal tubes and ovaries (Figure 4). The neonate was delivered with an APGAR score of 2 at one minute and 8 and five minutes. The neonate was evaluated by pediatrics who estimated her gestational age to be 36.5 weeks and she was well-appearing and transferred to the newborn nursery for routine neonatal care. Due to concerns about placental adherence to the bowel and solid organs, the placenta was left in situ. Interventional radiology was consulted for further angiographic imaging and possible placental embolization, which would allow the placenta to be removed in a delayed fashion with less risk of hemorrhage.

Following wound closure but prior to transfer from the OR, the patient became hemodynamically unstable with a heart rate of 160 and a blood pressure of 70/40. The operative team was concerned for occult or new-onset post-surgical bleeding or in-situ placental hemorrhage and the abdomen was re-entered. On reoperation, there was a large-volume hemoperitoneum and a supracervical hysterectomy was performed with placenta removal. During the initial operation, there was an estimated blood loss of 100 mL; however, with extensive hemoperitoneum present on the second operation, there was an estimated total blood loss of 3 L. She underwent a massive transfusion with a total of six units of packed red blood cells, six units of platelets, and one unit of fresh frozen plasma intraoperatively. She was transferred to the surgical intensive care unit (ICU) after the closure of her re-entry laparotomy. After one day in the ICU, she was extubated to a nasal cannula and then transferred to the floor the following day. The infant survived extraction, was admitted to the newborn nursery, and had no apparent complications. On postoperative day 3, the patient and infant were discharged home with scheduled outpatient follow-up.

## Discussion

AEP continues to represent a diagnostic and management challenge. The case discussed here demonstrates that clinical suspicion should remain high in all women of gestational age presenting with abdominal pain and that EP complications require expedited diagnosis and emergent specialty care.

The clinical presentation of AEP can be difficult to distinguish from other lower abdominal pathologies that present to the emergency department. Factors that should increase clinical concern for AEP include abdominal pain, peritonitis, lack of prenatal care, and suspected pregnancy without confirmed location on imaging [7]. Presentations remain variable and can present with recurrent abdominal pain, vomiting, painful fetal movements, palpable fetal parts, inability to establish fetal position, vaginal bleeding, as well as sonographic findings such as oligohydramnios, poor placental definition, or a uterus without amniotic sac and fetus [5,8,9]. Several risk factors should be considered when evaluating a patient's risk for abdominal pregnancy, including previous history of tubal pregnancies, pelvic inflammatory disease, tubal procedures, tubal infertility, and IUD use.

Early sonography is the mainstay of the diagnostic workup in such cases where EP or AEP is suspected. The reliability of ultrasound is linked inextricably with the expertise of both the clinical staff performing the ultrasound as well as the practitioner evaluating the imaging, which when performing bedside imaging is often the same individual [10]. Despite ultrasound’s high sensitivity, there have been reported cases of missed AEPs with multiple prenatal ultrasounds [5,8]. While ultrasound is the diagnostic modality of choice in the patient without confirmed intrauterine pregnancy due to its high sensitivity in early pregnancy, it may be necessary to obtain advanced imaging such as CT or magnetic resonance imaging (MRI) once the diagnosis is suspected to expedite surgical planning or to evaluate anatomic involvement in greater detail [4,8].

Ultrasound remains the gold standard in later gestational presentations, as it is often easily accessible and low cost; however, it can be difficult to diagnose ectopic or abdominal pregnancy later in gestation when pelvic organs are more difficult to visualize [7]. In the third trimester, ultrasound can be more difficult to interpret and sensitivity falls due to the large size of the fetus and positioning of the gravid uterus [10]. Clinicians must be careful not to develop overreliance on ultrasound alone to make the diagnosis of AEP, as a review of cases has demonstrated that even in early pregnancy, only 50% of early AEPs can be diagnosed on ultrasound alone [11]. In cases where ultrasound is indeterminate regarding pregnancy or placental location, it may become necessary to proceed with other imaging modalities to aid in the diagnosis. In stable patients with unclear ultrasound findings, MRI is often the next imaging modality obtained; however, if MRI is unavailable, there are concerns regarding patient hemodynamic stability, or the longer scan times of MRI are prohibitive, other imaging modalities can be used, including CT [9]. In the presented case, a CT was initially obtained, as the diagnosis of pregnancy was unknown and the patient had peritonitis on examination. There are also reported cases from low-resource settings lacking MRI and ultrasound where plain abdominal radiographs have been used to aid in making the diagnosis of AEP [12].

Once diagnosed, the management of AEP depends greatly on the stage at which it is diagnosed [9]. All cases require specialist obstetric care, and often, early laparotomy is required to eliminate the risk of placental abruption and life-threatening bleeding [9]. Some cases have been managed expectantly until planned laparotomy at 34 weeks gestation to allow the fetus to achieve lung maturity. However, with the expectant approach, there is a significant risk of hemorrhage and the patient should be kept under observation at a tertiary care facility where there are abundant resources and surgical staff present to manage complications [5,9]. The recommended medical management of the placenta in advanced cases remains debated and nuanced and relies on the evaluation of the blood supply to the placenta. Current recommendations include using a multidisciplinary team to cooperatively assess for embolization versus surgical resection of the placenta [11].

In cases where the placenta is left in situ, there is the risk of intra-abdominal hemorrhage after initial laparotomy, as seen in our case [11]. However, if there is no bleeding on examination during laparotomy and there is a desire to avoid the risk of further bleeding caused by surgical removal, the placenta can be left in situ and followed up to assess for self-absorption. The practice of administering methotrexate when leaving the placenta in situ remains controversial, as it can lead to severe intra-abdominal infection from rapid placental necrosis [11].

## Conclusions

AEP remains a rare and challenging diagnosis that may lead to significant maternal and fetal morbidity and mortality. Ectopic pregnancy should remain high on the differential for any patient with suspected pregnancy, especially those without routine prenatal care. Emergency physicians should continue to develop skills in obtaining and interpreting point-of-care ultrasound to increase diagnostic accuracy in both identifying the location of the pregnancy as well as any complications. Advanced imaging should be obtained if the ultrasound findings are indeterminate. Patients with AEP can decompensate rapidly and require expeditious specialty care. The medical and surgical management of abdominal pregnancy remains nuanced and cases require obstetric specialty care. The care plan should be determined individually with each presentation.
